# Optimizing Detection and Prediction of Cognitive Function in Multiple Sclerosis With Ambulatory Cognitive Tests: Protocol for the Longitudinal Observational CogDetect-MS Study

**DOI:** 10.2196/59876

**Published:** 2024-09-26

**Authors:** Anna Louise Kratz, Dawn M Ehde, Kevin N Alschuler, Kristen Pickup, Keara Ginell, Nora E Fritz

**Affiliations:** 1 Department of Physical Medicine and Rehabilitation University of Michigan Ann Arbor, MI United States; 2 Department of Rehabilitation Medicine University of Washington Seattle, WA United States; 3 Department of Neurology University of Washington Seattle, WA United States; 4 Department of Health Care Sciences Wayne State University Detroit, MI United States; 5 Department of Neurology Wayne State University Detroit, MI United States

**Keywords:** ambulatory assessment, longitudinal data collection, ecological momentary assessment, cognitive function, cognitive assessment, multiple sclerosis, physical activity, neuropsychology, physical function, observational study, wrist-worn accelerometry, social function, smartphone app, mobile phone

## Abstract

**Background:**

Cognitive dysfunction is a common problem in multiple sclerosis (MS). Progress toward understanding and treating cognitive dysfunction is thwarted by the limitations of traditional cognitive tests, which demonstrate poor sensitivity and ecological validity. Ambulatory methods of assessing cognitive function in the lived environment may improve the detection of subtle changes in cognitive function and the identification of predictors of cognitive changes and downstream effects of cognitive change on other functional domains.

**Objective:**

This paper describes the study design and protocol for the Optimizing Detection and Prediction of Cognitive Function in Multiple Sclerosis (CogDetect-MS) study, a 2-year longitudinal observational study designed to examine short- and long-term changes in cognition, predictors of cognitive change, and effects of cognitive change on social and physical function in MS.

**Methods:**

Participants—ambulatory adults with medically documented MS—are assessed over the course of 2 years on an annual basis (3 assessments: T1, T2, and T3). A comprehensive survey battery, in-laboratory cognitive and physical performance tests, and 14 days of ambulatory data collection are completed at each annual assessment. The 14-day ambulatory data collection includes continuous wrist-worn accelerometry (to measure daytime activity and sleep); ecological momentary assessments (real-time self-report) of somatic symptoms, mood, and contextual factors; and 2 brief, validated cognitive tests, administered by smartphone app 4 times per day. Our aim was to recruit 250 participants. To ensure standard test protocol administration, all examiners passed a rigorous examiner certification process. Planned analyses include (1) nonparametric 2-tailed *t* tests to compare in-person to ambulatory cognitive test scores; (2) mixed effects models to examine cognitive changes over time; (3) mixed effects multilevel models to evaluate whether ambulatory measures of physical activity, sleep, fatigue, pain, mood, and stress predict changes in objective or subjective measures of cognitive functioning; and (4) mixed effects multilevel models to examine whether ambulatory measures of cognitive functioning predict social and physical functioning over short (within-day) and long (over years) time frames.

**Results:**

The study was funded in August 2021 and approved by the University of Michigan Medical Institutional Review Board on January 27, 2022. A total of 274 adults with MS (first participant enrolled on May 12, 2022) have been recruited and provided T1 data. Follow-up data collection will continue through March 2026.

**Conclusions:**

Results from the CogDetect-MS study will shed new light on the temporal dynamics of cognitive function, somatic and mood symptoms, sleep, physical activity, and physical and social function. These insights have the potential to improve our understanding of changes in cognitive function in MS and enable us to generate new interventions to maintain or improve cognitive function in those with MS.

**Trial Registration:**

ClinicalTrials.gov NCT05252195; https://clinicaltrials.gov/study/NCT05252195

**International Registered Report Identifier (IRRID):**

DERR1-10.2196/59876

## Introduction

Multiple sclerosis (MS) is a chronic, inflammatory, autoimmune disease of the brain and spinal cord that affects approximately 1 million people in the United States [1]. It is the leading cause of nontraumatic disability in young adults [2]. Cognitive dysfunction is one of the most common problems in MS; up to 70% of people with MS report some type of cognitive dysfunction [3], including deficits in processing speed [4], episodic memory, visual memory, verbal fluency [5], working memory [6], and executive functioning [7]. Cognitive dysfunction in MS exerts a dire impact on many aspects of health-related quality of life, including employment, independent living, social participation, and physical functioning [8-10], and has been linked to poor treatment adherence [11]. Unfortunately, progress in developing preventative, compensatory, and restorative interventions for cognition in MS is stymied by major gaps in our knowledge of the natural history of cognitive decline and of the characteristics and mechanisms of cognitive dysfunction where it matters most—in the everyday lives of people with MS [12].

Our knowledge of the nature and impact of cognitive functioning in MS is restricted by measurement limitations and insufficient attention to potential contributors to and consequences of changes in cognition. Measurement of cognitive function is limited by reliance on cross-sectional data and the use of standard neuropsychological testing protocols. These assessment protocols are insensitive to subtle cognitive changes and demonstrate practice effects, temporal bias, and poor ecological validity [13-15]. A crucial limitation is that the “snapshot” of cognitive function, typical of cross-sectional neuropsychology studies, fails to capture day-to-day and within-day variability in an individual’s cognitive function [16,17]. Understanding such short-term variability in cognitive function in MS is important for 3 key reasons: first, within-person fluctuations in cognitive performance may be an independent indicator of poor cognitive functioning [18,19] and vulnerability to future cognitive decline [20,21]. Second, identifying time-varying modifiable factors that precede and predict changes in cognitive dysfunction can provide crucial clues about potentially productive targets for intervention, particularly real-time interventions that can have immediate effects. Finally, studying within-person covariation between cognitive function and other functional domains, such as social and physical function, can provide convincing evidence as to the contribution of cognitive dysfunction to important person-centered outcomes.

To address measurement limitations of gold-standard neuropsychological testing, this study leverages technology-assisted ambulatory assessment techniques to provide a unique and multidimensional window into cognitive dysfunction in the everyday lives of people with MS. Multiple complementary ambulatory assessment strategies are used. A customized smartphone app is used to administer a battery of objective ambulatory cognitive tests that are designed specifically for serial administration in the lived environment, as well as ecological momentary assessments (real-time assessment) of self-reported symptoms and functioning as a person goes about daily life, an approach that is not as subject to recall bias or memory decay [22]. The smartphone app is paired with accelerometer technology, which provides objective, continuous, and unobtrusive measures of physical activity during day and night (ie, sleep). Ambulatory assessments are administered in a “measurement burst design,” incorporating bursts of intensive repeated assessment in people with MS over 2 weeks, with bursts repeated longitudinally, at baseline and 1- and 2-year follow-up. The burst design provides 2 main benefits: improved detection of subtle long-term changes in cognitive functioning and the ability to examine fine-grained temporal associations between fluctuations in daily experiences (eg, pain, fatigue, and stress) and cognitive function [23].

Using these innovative assessment methods, we aim to explore foundational questions that have yet to be examined in MS, such as the degree and prognostic utility of within-person lability in cognitive function. We will determine if ambulatory assessments are sensitive to subtle declines in cognitive functioning. We will also explore the impact of modifiable factors, such as sleep, physical activity, mood, and somatic symptoms on cognitive function. Finally, we will explore whether variability in cognitive functioning predicts short- and long-term changes in other patient-centered functional domains, social participation, and physical functioning. In pursuit of these primary objectives, the study is designed to test three hypotheses: (1) ambulatory measures of subjective and objective cognitive function will be more sensitive to longitudinal changes (over 2 years) in cognitive functioning compared to conventional clinic-based assessments; (2) ambulatory measures of modifiable factors—physical activity, sleep, fatigue, pain, mood, and stress—predict short-term (same-day) and long-term (at 1- and 2-year follow-up) changes in cognitive functioning; and (3) ambulatory measures of cognitive functioning will predict social and physical functioning over short- (same-day) and long-term (at 1- and 2-year follow-up) time frames.

## Methods

### Study Design

The Optimizing Detection and Prediction of Cognitive Function in Multiple Sclerosis (CogDetect-MS) study applies an observational design that combines microlongitudinal (ie, frequent, repeated “burst” measures across 14 consecutive days) and longitudinal (ie, 1- and 2-year follow-up) data collection methods in a sample with MS. Participant recruitment and data collection are conducted across 3 sites: the University of Michigan (UM; lead site and data coordinating center) in Ann Arbor, Michigan; Wayne State University (WSU) in Detroit, Michigan; and the University of Washington (UW) in Seattle, Washington. Ambulatory data are managed by researchers at the Pennsylvania State University, who return scored and combined ambulatory datasets to UM.

### Ethical Considerations

This multisite study has received a single institutional review board (IRB) approval from the medical IRB at UM (HUM00199732; participating site approvals: UM: HUM00213744, WSU: SITE00000462, and UW: SITE00000461). Initial IRB approval was obtained on January 27, 2022. All volunteers provided written informed consent. Participants are compensated US $600 for the full completion of the study (US $200 for each visit—T1, T2, and T3). For those who do not complete the full study, the compensation schedule is as follows: US $50 per laboratory visit and US $150 per home monitoring period (for <14 days of data, compensation is graded with US $4 per day for days 1-5, US $10 per day for days 6-10, and US $20 per day for days 11-14).

### Study Sample and Recruitment

The aim was to recruit 250 participants, with the expectation that 210 would also provide data at the final (2-year) follow-up. Participants were recruited through existing participant registries; electronic health record queries; institution-specific, participant-recruitment websites; clinic- and community-based recruitment; posting of flyers; and outreach to local partners, such as the local chapters of the National MS Society. Inclusion criteria (assessed by self-report) were (1) 18 years of age or older, (2) able to fluently converse and read in English, (3) MS diagnosis (confirmed via medical record review; all relapsing and progressive subtypes included), and (4) able to ambulate either independently or with the use of a cane or walker (or similar device) for at least 50% of the time at baseline; participants who lose ability to ambulate over the course of the study are retained, as this criteria only applies to initial enrollment. Exclusion criteria were (1) MS relapse within the past 30 days (may become eligible after 30 days; criteria used at T1, T2, and T3) and (2) inability to use study data collection tools (ie, ActiGraph wGT3X-BT [ActiGraph], smartphone app; volunteers “pass” this final exclusion criterion by independently completing a trial of the ambulatory assessment battery during the laboratory visit).

### Participant Screening, Enrollment, and Data Collection Procedures

Volunteers underwent an initial prescreening by telephone to determine general inclusion or exclusion criteria and were fully screened at the T1 laboratory visit to establish study eligibility. MS diagnosis was either preconfirmed through medical record review or initially gathered by self-report and later confirmed through record review. Written informed consent procedures were either conducted remotely (via Zoom [Zoom Video Communications] or telephone, and a signature obtained via e-consent in REDCap [Research Electronic Data Capture; Vanderbilt University]) prior to the T1 laboratory visit or in person at the laboratory visit.

Participation in this study involves assessments at baseline (T1), 1-year follow-up (T2), and 2-year follow-up (T3). Each assessment period includes a ~2.5-hour laboratory visit immediately followed by a 14-day ambulatory monitoring period (measurement “burst”). At each laboratory visit, certified examiners administer cognitive and physical function test batteries and demonstrate the use of a study-specific smartphone (programmed with a data collection app) and an ActiGraph wGT3X-BT accelerometer for the collection of ambulatory data. A battery of web-based self-report surveys is also completed at each time point, either prior to (within 30 days of laboratory visit) or during the laboratory visit.

During each ambulatory monitoring period, participants continuously wear an ActiGraph wGT3X-BT to passively collect physical activity data. At 4 intervals throughout the day (wake, midday, and bedtime), participants complete a set of brief, valid, and reliable cognitive tests assessing processing speed and working memory [13] along with a battery of ecological momentary assessment (EMA; real-time self-report) measures of somatic symptoms, mood, functioning, behaviors, and context on the smartphone app. The wake-up and bedtime assessments are initiated by the participant when waking up (ie, waking and not necessarily getting out of bed) and going to bed (ie, “lights out” and not necessarily when getting into bed). The other 2 assessments are prompted by an audible alert on a quasi-random schedule determined by their usual waking time. At the end of each home monitoring period, participants return the ActiGraph wGT3X-BT and smartphone in a prepaid mailer to the laboratory for data download.

### Data Collection Platforms and Technology

Survey data are collected via a secure, study-specific REDCap website. REDCap is an open-source, secure, HIPAA (Health Insurance Portability and Accountability Act)–compliant, web-based platform designed to support data capture for research studies. It has been designed specifically to protect patient privacy and confidentiality while assisting investigators in clinical research. REDCap provides an interface for data entry and validation, auditing features for tracking data manipulation, the ability to import data from external sources, calculated data fields, branching logic, and the capability to export data to many statistical packages. System-level and app-level security include Secure Sockets Layer encryption of internet traffic (https pages), hosting in a secure data center with nightly backup, fine-grained control over user rights, detailed audit trails, record locking, and deidentification features for data export. REDCap was initially developed by Vanderbilt University but now has collaborative support from a wide consortium of many domestic and international partners.

**Figure 1:**
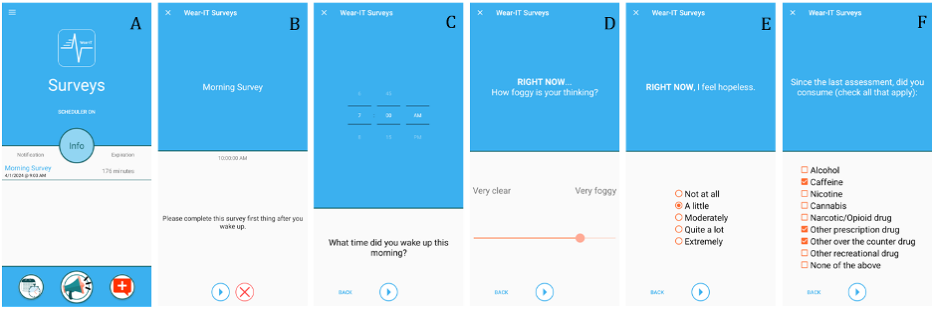
(A) The Wear-IT app landing page, (B) morning survey landing page, (C) self-reported wake-up time, (D) an item from the perceived cognitive function scale, (E) an item from the depressed mood scale, and (F) the survey of substance consumption.

Self-report EMAs and ambulatory cognitive tests are administered via a customized app (Figure 1; Wear-IT, developed by the Real Time Science Lab, Pennsylvania State University) installed on a Motorola g^8^ Power mobile phone with a 6.4″ display (1080×2300 pixels). The phone is loaned to participants for use during the study; it is not associated with any phone number and is used with an inactive SIM card for keeping accurate time on the phone; thus, there are no signals sent or received via the phone. Phones are loaned to study participants to ensure device consistency across participants and across time periods and because ambulatory cognitive assessment apps on personal phones have not been validated at the time the study launched. Response times are recorded in milliseconds. Data are stored onboard the smartphone until it is returned to the laboratory for data download.

**Figure 2:**
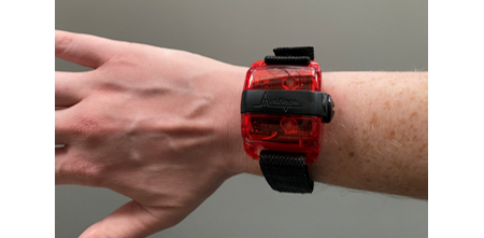
The ActiGraph wGT3X-BT triaxial accelerometer.

The ActiGraph wGT3X-BT triaxial accelerometer (Figure 2) is used to measure physical activity. It is mounted on a fabric band on the nondominant wrist. In cases of hemiparesis, the accelerometer is placed on the nonparetic side. It is lightweight (19 g) and compact (3.3×4.6×1.5 cm) and measures movement using a capacitive accelerometer that digitizes a voltage detected from movement at a sampling rate of 30 Hz. The samples are summed over a 60-second epoch period and output as activity counts. Higher activity counts relate to more physical activity. We use a wrist-worn placement, as this placement has been used extensively in physical activity studies and to validate the ActiGraph in MS [24-35].

Smartphone app data (EMA and cognitive tests) are combined, and time synced with accelerometer date by the Wear-IT team.

### CogDetect-MS Study Measures: Laboratory Visit Measures

#### Self-Report Measures

The self-report survey battery includes surveys of demographics, clinical characteristics, and medical history (eg, medications and therapies) and a selection of valid and reliable self-report measures (Table 1 and Multimedia Appendix 1).

**Table 1:** Validated self-report surveys administered in the Optimizing Detection and Prediction of Cognitive Function in Multiple Sclerosis (CogDetect-MS) study.

Domain	Measures
Sleep	PROMIS^a^ Sleep Disturbance Short Form 8b [36] STOP-Bang [37]
Fatigue	PROMIS Fatigue Short Form 8 V1.0 [38] Michigan Fatigability Index Short Forms
Pain	PROMIS Pain Intensity 3a [36] PROMIS Pain Interference 8a [39-41] painDETECT [42] American College of Rheumatology Fibromyalgia Diagnostic Criteria [43,44]
Depressed mood	PROMIS Depression 8b [45]
Stress	Perceived Stress Scale [46]
Social functioning	Neuro-QoL Ability to Participate in Social Roles and Activities Short Form 8 [47,48]
Cognitive function	PROMIS Cognitive Abilities Short Form 8 [49] Compensatory Cognitive Strategies Scale [50]
Physical functioning	Neuro-QoL Upper Extremity Function-8 [47] Neuro-QoL Lower Extremity Function-8 [47] Patient Determined Disease Steps [51]
Falls	1-Month Falls History Falls Efficacy Scale [52] Fear of Falling Avoidance Behavior Questionnaire [53] Concern and Fear of Falling Evaluation [54,55]
Substance use	Tobacco, Alcohol, Prescription Medications, and Other Substances Tool [56-58]
Comorbidities	Comorbidity Questionnaire [59]
Personality^b^	Ten-Item Personality Inventory [60]
Demographic and clinical variables	Demographic and clinical characteristics survey Pet ownership survey [61]

^a^PROMIS: Patient Reported Outcomes Measurement Information System.

^b^Evaluated at T1 visit only.

#### Performance-Based Laboratory Measures

##### Motor Function

We administer the full lower-extremity and upper-extremity National Institutes of Health Toolbox (NIHTB) motor test battery [62,63] via the NIHTB iPad App. In addition to the NIHTB motor measures, we also administer a 4-Meter Backward Walking Test to calculate backward walking speed [54,64,65]. Table 2 provides a full list of motor tests, and Multimedia Appendix 2 provides further details.

**Table 2:** Cognitive and physical performance tests administered in the Optimizing Detection and Prediction of Cognitive Function in Multiple Sclerosis (CogDetect-MS) study.

Domain	Measures
Cognitive function	Symbol Digit Modalities Test (oral administration) [66] Paced Auditory Serial Addition Test-3 seconds [67]^a^ Rey Auditory Verbal Learning Test [68]^a^ ReacStick Test [69] NIH^b^ Toolbox Cognitive Battery [62,70]: Dimensional Change Card Sort Test Flanker Inhibitory Control and Attention Test List Sorting Working Memory Test Oral Reading Recognition Test Oral Symbol Digit Test Pattern Comparison Processing Speed Test Picture Sequence Test^a^ Picture Vocabulary Test
Physical function	4-Meter Backward Walking Test [64] NIH Toolbox Motor Battery [62,63] 2-Minute Walk Endurance Test 4-Meter Walk Gait Speed Test 9-Hole Pegboard Dexterity Test Grip Strength Test Standing Balance Test

^a^Alternate test forms used across T1, T2, and T3.

^b^NIH: National Institutes of Health.

##### Cognitive Function

We administer the NIHTB cognitive test battery plus the supplemental NIHTB Oral Symbol Digit Test [62,70] via the NIHTB iPad App. We also administer the Symbol Digit Modalities Test (oral administration) [66], the Paced Auditory Serial Addition Test (3 seconds) [67], the Rey Auditory Verbal Learning Test [68], and the ReacStick Test [69]. See Table 2 for a full list of laboratory-based cognitive tests and Multimedia Appendix 2 for further details.

#### Ambulatory Measures

A set of EMA items and scales (administered via a smartphone app, 4× per day except where noted) are administered. Some measures were adapted for daily administration from existing validated recall measures. See Table 3 for a full list of items and Multimedia Appendix 3 for further details.

**Table 3:** Ambulatory data collected in the Optimizing Detection and Prediction of Cognitive Function in Multiple Sclerosis (CogDetect-MS) study.

Data type and measure			Schedule
**Ecological momentary assessment (via smartphone app)**			
	Perceived cognitive function (3 items)	4× per day	
	Pain intensity (1 item)	4× per day	
	Fatigue intensity (2 items)	4× per day	
	Perceived stress (1 item)	4× per day	
	Depressed mood (3 items)	4× per day	
	Location during cognitive tests (1 item)	4× per day	
	Distractions during tests (4 items with branching logic)	4× per day	
	Substance use (1 item)	4× per day	
	Activity pacing (3 items)	3× per day^a^	
	Sleep quality (2 items)	Morning	
	Overnight falls (4 items with branching logic)	Morning	
	Social participation (6 items)	Evening	
	Physical function (2 items)	Evening	
	Daytime falls (4 items with branching logic)	Evening	
**Cognitive function (via smartphone app)**			
	Symbol Search Test	4× per day	
	Dot Memory Test	4× per day	
**Physical activity (via ActiGraph accelerometer)**			
	Daytime physical activity and nighttime sleep activity	Continuous for 24 hours	

^a^All time points except morning.

**Figure 3:**
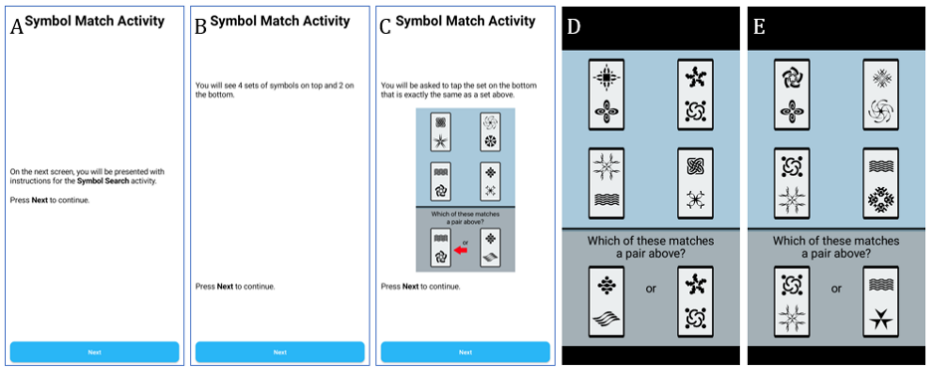
(A) Symbol Search landing page, (B and C) test instructions, and examples of (D) a “nonlure trial” (where neither symbol in the incorrect pair on the bottom appears in the pairs above) and (E) a “lure trial” (where one of the symbols in the incorrect pair on the bottom appears in a pair above).

Two brief, valid, and reliable cognitive tests [13] are administered via the smartphone app. Response time speed is recorded in milliseconds for all tests. The Symbol Search Test (Figure 3) is a test of processing speed. Participants see a 2x2 grid of 4 symbol pairs at the top of the screen and are presented with 2 symbol pairs at the bottom of the screen. Stimuli are presented until a response is provided. Participants decide, as quickly as possible, which symbol pair at the bottom matches one of the symbol pairs at the top and select the matching pair by touching their selection at the bottom. In total, 24 trials are administered for each session. Reaction time and errors are recorded for sessions where effort is deemed adequate (accuracy >70%).

**Figure 4:**
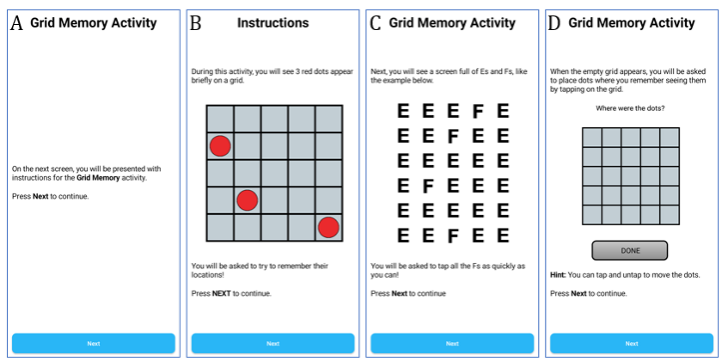
(A) Dot Memory Test landing page, (B) instructions, (C) E’s and F’s distraction phase, and (D) response page.

The Dot Memory Test (Figure 4) is a test of working memory. Each trial consists of 3 phases: encoding, distraction, and retrieval. During the encoding phase, the participant is asked to remember the location of 3 red dots appearing on a 5×5 square grid. After a 3-second study period, the grid is removed, and the distraction phase begins, during which the participant is required to locate and touch the F’s in an array of E’s. After performing the distraction task, an empty 5×5 square grid is presented, and the participant must place the red dots (by touching the empty squares) in the correct locations. Participants press “Done” when they are finished. Speed and Euclidean distance (a score of the collective distance of the 3 dots from their correct locations) are recorded. In total, 4 trials are administered for each session.

The ActiGraph produces variables representing different facets of day and nighttime physical activity. Our main measurements for daytime (awake) activity are activity counts, steps, physical activity intensity, and sedentary bouts across the 14-day home monitoring period, providing daily and typical activity levels. Our main measures for nighttime activity will be sleep latency, total sleep time, wake after sleep onset, and sleep efficiency.

### Examiner Certification Process

To ensure standard test protocol administration across study sites and time, a rigorous examiner certification process was established. To achieve initial certification to administer tests, research staff were required to read the study protocol and manual of procedures, read the NIHTB Administration Manual, watch all training videos, and pass quizzes at the end of each training video. Training videos were from the NIH Toolbox eLearning Course or were custom-made by the study investigators, who had expertise in motor testing (NEF) or neuropsychological test administration (ALK, DME, and KNA). The videos provide detailed instructions on general best practices for test administration and how to administer each non-NIHTB test. After these initial training activities, research staff practiced the full laboratory-visit protocol with at least 5 nonparticipants (eg, fellow laboratory staff), video recording the final testing session. This video along with all accompanying case report forms and test materials were evaluated by 2 investigators—one with expertise in administering motor tests and one with expertise in administering neuropsychological tests. Together, the evaluators decided whether the examiner passed or failed the certification. Failure is defined as 2 or more minor errors or 1 or more major errors. A major error is defined as any error that indicates a lack of understanding of the proper standardized administration of any test or any scoring error that is large enough to change the interpretation of the data. Errors are reviewed with the examiner and their site principal investigator (ALK, NEF, or KNA). If the assessment is failed, the examiner practices at least 1 more time and submits a new certification video for review; this process can continue until the examiner passes certification. After initial certification, the examiner can begin testing study participants and is required to video record the first testing session with a person with MS; this video is also reviewed for consistency with study protocol, and feedback shared with the examiner. To ensure continued adherence to testing protocol, examiners record the laboratory visit for every 10th session, and this recording is reviewed by investigators as with the earlier certification videos. Consent for video recording is included in the study consent form.

### Data Monitoring

The principal investigators (ALK and NEF) and lead research coordinator (KP) from the data coordinating center (UM) conduct in-person data audits at each site on an annual basis. Data related to adverse events, protocol deviations, study personnel training, screening procedures, participant withdrawal or termination, and enrollment procedures and documentation were audited for all participants enrolled at the site; data related to eligibility screening and documentation, study visit tracking, participant contact information, compensation record of human participants, and data collection were audited for a random subsample of all participants enrolled at the site. Audit reports, detailing findings, and required responses to the audit were produced and delivered to the site principal investigator (ALK, NEF, or KNA) and lead site research coordinator.

### Multisite Coordination

To ensure multisite coordination and fidelity of procedures, the full study team, including all examiners and investigators at all study sites, met weekly to discuss study-related questions and troubleshoot any issues that had arisen during the prior week for the first 18 months of the study. As fewer questions arose, and study teams were immersed in recruitment and testing, meetings were shifted to every other week (months 18-36). After month 36, meetings were shifted to once per month. UM keeps a record of all meeting agendas and meeting minutes, and any clarifications to the study manual of procedures are recorded by the UM team and updated in a shared folder that includes all study-related documents.

UM serves as the data coordinating center for the study. Data from all sites are fully accessible to the investigators and staff at UM, who conduct monthly data checks to assess for data completeness and quality. Data double entry of case report forms from each study site and data cleaning, scoring, and merging to produce final, analyzable datasets are completed by UM staff and investigators.

### Sample Size Analyses

We conducted analyses to determine the sample size needed to address all study aims. The goal of the first study aim is to determine whether the ambulatory tests are able to detect cognitive decline from baseline (T1) to 1-year (T2) or 2-year follow-up (T3) for individuals where clinic-based tests do not detect decline. The proportion of participants who show no decline or improvement, absolute but subtle decline (change <1/2 SD), meaningful decline (change between 1/2 SD-1 SD) [71], or clinically significant decline (≥1 SD decline) [71,72] will be calculated for both ambulatory and clinic-based neurocognitive tests. For each cognitive domain, a binary variable will be created for each participant indicating whether the ambulatory and clinic-based cognitive measures are consistent with each other (eg, agree) about the degree of change or laboratory-based or ambulatory measures indicate a larger degree of decline. We will test whether the proportion of cases where ambulatory measures indicated a larger degree of decline (relative to clinic-based tests) is statistically different from 0; sample size analyses for this test indicate that a sample of 199 will have 95% power (with critical α=.01) to detect significance, where ambulatory cognitive tests show a greater level of decline compared to clinic-based tests in as few as 1.5% (n=3) of cases. This suggests that our expected final sample size of 210 has the power to detect even modest differences in analyses comparing proportions of the sample that show a decline on ambulatory cognitive tests but not on clinic-based tests.

Effects sizes from a prior study of perceived cognitive functioning in daily life in MS [73-75] informed sample size estimation for the second and third aims, which examines factors that predict later cognitive decline or that are predicted by cognitive changes. We calculated the sample size needed to test the aims in a linear regression framework [76], which is a relatively conservative estimate, given that the repeated measures design imparts greater measurement reliability and therefore greater power [77]. We based our estimates on models that included up to 6 covariates (see list of covariates below) and 6 predictor variables of interest (eg, sleep quality, physical activity, pain, fatigue, mood, and stress) in predicting any given cognitive variable. The sample size for these models was expected to provide a conservative estimate for power required for the third aim (which had fewer predictors in each model). Critical α (*P*) value was set at .01. Our analyses indicated that a sample size of 214 will have 95% power (critical *t*=2.34, 1-sided significance test) to detect an association between cognitive functioning, and the variable expected to show the weakest association with cognition: mood (effect size *f*^2^=0.075).

### Data Analysis

#### Overview

Primary data analyses will account for covariates that have been shown to be associated with cognitive change in MS—age, sex, disease duration, MS subtype (relapsing vs progressive subtypes combined), disease severity, personality variables, and cognitive reserve (education level plus scores on vocabulary test). After primary analyses are completed, analyses will be repeated stratifying by sex, age group, baseline cognitive impairment, and MS subtype. We have intentionally included participants with both existing cognitive impairment and no known cognitive impairment at enrollment. This will allow us to also conduct sensitivity analyses to explore whether people who show evidence of cognitive impairment at baseline show a more rapid decline on either laboratory or ambulatory cognitive assessments as has been identified in prior research [78,79].

#### Specific Aim 1: Are Ambulatory Measures of Subjective and Objective Cognitive Function More Sensitive to Longitudinal Changes in Cognitive Function Compared With Conventional Clinic-Based Assessments?

Nonparametric tests (Wilcoxon signed rank test, 2-tailed) will be used to compare cognitive test scores (ambulatory vs clinic-based measures) for each participant at each time point. We will test whether the proportion of cases where the ambulatory test indicates a greater degree of cognitive decline compared to the clinic-based cognitive tests is statistically different from 0. Additional sensitivity tests of the paired differences in proportions of 4 categories (no decline, absolute but subtle decline, meaningful decline, or clinically significant decline) for each cognitive domain between ambulatory and clinic-based neurocognitive tests at both 1- and 2-year follow-up will be conducted [80]. Mixed effects models will be used to examine changes over time for each cognitive measure, with the expectation that the ambulatory measures will show larger time effects at both 1- and 2-year follow-up.

#### Specific Aim 2: Do Modifiable Factors Predict Short- and Long-Term Changes in Ambulatory Measures of Cognitive Functioning?

##### Short Term

Mixed effects multilevel models (MLMs) for momentary (within-day) associations, one for each ambulatory cognitive variable (perceived cognitive function and cognitive test scores), will be constructed. In each case, predictor variables of interest will be physical activity (accelerometer data), sleep (accelerometer data and EMA-sleep quality), and EMA measures of fatigue, pain, mood, and stress from the previous within-day time point (all moment-to-moment analyses will be conducted within-day). Similarly, MLMs for day-level associations, one for each cognitive variable, will be constructed; only day-level analyses will explore the association between sleep and cognition. Given the lack of data on the temporal effects of these variables on cognitive functioning, exploratory analyses of lagged effects (1- and 2-day lag) will also be examined.

##### Long Term

Ambulatory measures of predictor and outcome variables will be aggregated within a time period for baseline and 1- and 2-year follow-up periods. Laboratory-based measures of cognitive functioning will also be examined. MLMs will be used to test whether ambulatory measures of physical activity, sleep, fatigue, pain, mood, and stress predict changes in objective or subjective measures of cognitive functioning (ambulatory and laboratory-based measures) 1 or 2 years later. The change will be modeled within an analysis of covariance framework, where T2/T3 values for an outcome of interest are modeled controlling for T1 values of the said outcome. In contrast to specific aim 1, where the primary interest is on comparing the performance of the ambulatory tests to standard clinic-based cognitive tests, the primary interest of specific aim 2 is in understanding what factors contribute to variation or changes in cognitive function and in identifying probable targets for cognitive rehabilitation regardless of the measure used to identify such associations; therefore, no direct comparisons between measurement types will be made.

#### Specific Aim 3: Do Ambulatory Measures of Cognitive Functioning Predict Social and Physical Functioning Over Short-Term and Long-Term Time Frames?

##### Short Term

In the momentary data, MLMs will be constructed to predict same-day social participation and physical functioning (upper- or lower-extremity functioning, balance, and falls or missteps) from the ambulatory cognitive variables. Analyses for falls or near falls will be conducted using a special case of MLM for categorical outcomes. Exploratory analyses of lagged effects (1- and 2-day lag) will also be examined.

##### Long Term

In terms of distal prediction of social and physical function from ambulatory cognition, we will conduct MLMs with the cognitive variables (averaged across each time period) predicting social and physical functioning at 1- and 2-year follow-up. The change will be modeled within an analysis of covariance framework, where T2/T3 values for an outcome of interest are modeled controlling for T1 values of the said outcome. These MLMs exploring long-term associations between cognitive changes and changes in social and physical functioning will be repeated in a set of secondary analyses with standard clinic-based cognitive test scores as predictor variables. Prediction of long-term changes in social and physical functioning from ambulatory cognitive measures will be compared to the ability of laboratory-based measures to predict these same changes. Ambulatory measures of cognition are of primary interest for specific aim 3, given that their microlongitudinal burst design allows for examination of short- and long-term associations, and are assumed to be more reliable and therefore more likely to demonstrate robust associations with the other functional outcomes.

## Results

This research received funding on August 1, 2021, from the Eunice Kennedy Shriver National Institute of Child Health & Human Development (R01HD102337-01A1). Enrollment and T1 data collection occurred between May 12, 2022, and February 29, 2024. The study recruited 301 individuals with MS (UM: n=107, WSU: n=102, and UW: n=92); of these, 274 (UM: n=101, WSU: n=88, and UW: n=85) participated in T1 data collection. Longitudinal data collection will continue through March 2026. Data analysis has not yet started as of the time of this submission.

## Discussion

### Principal Findings

The anticipated findings of this study are 3-fold. First, we anticipate that ambulatory cognitive tests will be more sensitive to subtle cognitive changes in people with MS over 1-2 years. Second, we expect to identify modifiable factors (eg, mood, sleep, and physical activity) that precede and predict later cognitive decline on a short- and long-term scale. This information could help to prevent future decline or mitigate current cognitive dysfunction. Third, we hypothesize that cognitive changes will predict changes in social and physical function on a short- and long-term scale; such information will help to delineate the full impact of cognitive change in MS.

The collection of intensive longitudinal data in a large, heterogenous sample will allow for an in-depth characterization of individuals with MS and provide multiple avenues for future research. Data from this study are expected to inform comprehensive models of cognitive change in MS as well as provide insights on potential targets for intervention development to help people with MS optimize cognitive function.

### Strengths and Limitations

One limitation of this study is that the inclusion criteria require participants to be able to ambulate. The rationale for this criterion is that we would like to collect meaningful accelerometer data in order to explore the associations between physical activity and cognitive function. However, this criterion limits the generalizability of the findings to those with more significant mobility limitations.

This study has a number of notable strengths. Technology-enabled assessment of day-to-day cognitive function in the lived environment has the potential to greatly improve the sensitivity and ecological validity of cognitive assessment in people with MS. The advancement of measurement sensitivity is critical, as cognitive changes can be subtle and compound slowly over time; however, despite the small magnitude of these changes, individuals with MS often report distress over noticeable changes in their cognition that are not detected on standard laboratory-based cognitive tests. Another advantage is the intensive within-person design that allows for the exploration of dynamic associations between potentially modifiable predictors of cognitive dysfunction while accounting for “third variables” such as a person’s disease severity, age, and sex. AnΩΩΩhone. This allows for exploring different trajectories of change over time.

### Future Directions

Future work that capitalizes on the findings of this study and advances in assessment methods can also be used to improve treatment decision-making, including the timing and type of treatment approach. We will use these findings to design and test trials of behavioral, medical, and combination therapies to prevent cognitive decline and improve cognitive functioning in people with MS. Additionally, our innovative assessment methods may also be used to improve the measurement of outcomes in clinical trials of both pharmacological and nonpharmacological interventions, enriching understanding of the effects of such interventions. To our knowledge, there are few studies that track cognitive and other functional domains in MS beyond 2 years; thus, long-term outcomes will yield a rich dataset for understanding longitudinal function in persons with MS.

## Appendix

Multimedia Appendix 1 Detailed description of CogDetect-MS self-report measures. CogDetect-MS: Optimizing Detection and Prediction of Cognitive Function in Multiple Sclerosis.

Multimedia Appendix 2 Detailed description of CogDetect-MS performance-based measures. CogDetect-MS: Optimizing Detection and Prediction of Cognitive Function in Multiple Sclerosis.

Multimedia Appendix 3 Detailed description of CogDetect-MS ambulatory self-report measures. CogDetect-MS: Optimizing Detection and Prediction of Cognitive Function in Multiple Sclerosis.

## Abbreviations

CogDetect-MS: Optimizing Detection and Prediction of Cognitive Function in Multiple Sclerosis
EMA: ecological momentary assessment
HIPAA: Health Insurance Portability and Accountability Act
IRB: institutional review board
MLM: multilevel model
MS: multiple sclerosis
NIHTB: National Institutes of Health Toolbox
REDCap: Research Electronic Data Capture
UM: University of Michigan
UW: University of Washington
WSU: Wayne State University

## Data Availability

The datasets generated during this study are not publicly available at the time of this publication due to ongoing data collection but will be available at the conclusion of the study on a secure web-accessible data repository. The availability of data sharing will be publicized in publications and presentations.
