# Supplementary material for: Optimizing Detection and Prediction of Cognitive Function in Multiple Sclerosis With Ambulatory Cognitive Tests: Protocol for the Longitudinal Observational CogDetect-MS Study
Source: JMIR Res Protoc. 2024 Sep 26;13:e59876. doi: 10.2196/59876 (PMC11467611; doi:10.2196/59876)
Supplement: Multimedia Appendix 1 [file resprot_v13i1e59876_app1.docx]

- Substance use: An adapted form of the Tobacco, Alcohol, Prescription Medications, and Other Substances (TAPs) Tool was used to assess substance use. The TAPS Tool was adapted to assess cannabis use independent of illegal/recreational drug use because cannabis is currently legal for recreational use in both states in which the study is being conducted and cannabis is often used medicinally in MS.
- PROMIS Pain Interference 8a: This 8-item short form assesses self-reported consequences of pain on relevant aspects of one’s life. This includes the extent to which pain hinders engagement with social, cognitive, emotional, physical, and recreational activities. This measure is scored on a T-score metric (with a mean of 50 and standard deviation of 10); the referent population is the general population. Higher scores indicate more pain interference. Reliability and validity data support the clinical utility of this measure.
- PROMIS Pain Intensity Short Form: This 3-item short form assesses how much a person hurts. The first two items assess pain over the past seven days while the last item assesses current pain intensity. This measure is scored on a T-score metric (with a mean of 50 and standard deviation of 10); the referent population is the general population. Higher scores indicate more intense pain. Reliability and validity data support the clinical utility of this measure.
- PROMIS Cognitive Abilities Short Form: This 8-item short form assesses patient-perceived cognitive function. This measure is scored on a T-score metric (with a mean of 50 and standard deviation of 10); the referent population is the general population. Higher scores indicate greater cognitive functioning. Reliability and validity data support the clinical utility of this measure.
- Neuro-QoL Lower Extremity Functioning Short Form: This 8-item short form assesses one’s ability to carry out various activities involving the trunk region and increasing degrees of bodily movement, ambulation, balance or endurance. This measure is scored on a T-score metric (with a mean of 50 and standard deviation of 10). Higher scores indicate better ability. Reliability and validity data support the clinical utility of this measure.
- Neuro-QoL Upper Extremity Functioning Short Form: This 8-item short form assesses one's ability to carry out various activities involving digital, manual and reach-related functions, ranging from fine motor to self-care (activities of daily living). This measure is scored on a T-score metric (with a mean of 50 and standard deviation of 10). Higher scores indicate better ability. Reliability and validity data support the clinical utility of this measure.
- PROMIS Depression Short Form: This 8-item short form assesses self-reported negative mood (sadness, guilt), views of self (self-criticism, worthlessness), and social cognition (loneliness, interpersonal alienation), as well as decreased positive affect and engagement (loss of interest, meaning, and purpose). This measure is scored on a T-score metric (with a mean of 50 and standard deviation of 10); the referent population is the general population. Higher scores indicate more depression. Reliability and validity data support the clinical utility of this measure.
- PROMIS Fatigue Short Form: This 7-item short form assesses a range of self-reported symptoms, from mild subjective feelings of tiredness to an overwhelming, debilitating, and sustained sense of exhaustion that likely decreases one’s ability to execute daily activities and function normally in family or social roles. The referent population is the general population. Higher scores indicate more fatigue. Reliability and validity data support the clinical utility of this measure.
- Michigan Fatigability Index (MIFI) Fatigability Short Forms: Three 6-item short forms assess physical fatigability, mental fatigability, and emotional fatigability. Higher scores indicate greater fatigability.
- PROMIS Sleep Disturbance Short Form: This 8-item short form assesses self-reported perceptions of sleep quality, sleep depth, and restoration associated with sleep. This includes perceived difficulties and concerns with getting to sleep or staying asleep, as well as perceptions of the adequacy of and satisfaction with sleep. Higher scores indicate more sleep disturbance. The final score is represented by the T-score, a standardized score with a mean of 50 and a standard deviation (SD) of 10. Reliability and validity data support the clinical utility of this measure.
- Neuro-QoL Ability to Participate in Social Roles & Activities Short Form: This 8-item short form assesses a person’s ability to participate in various social roles and activities across a range of social domains (home/family, recreation).  Higher scores indicate better ability to participate. This measure is scored on a T-score metric (with a mean of 50 and standard deviation of 10). Reliability and validity data support the clinical utility of this measure.
- Ten-Item Personality Inventory (TIPI): This10-item measure assesses the Big Five (or Five-Factor Model) dimensions. This measure reached adequate levels in terms of (a) convergence with widely used Big-Five measures in self, observer, and peer reports, (b) test-retest reliability, c) patterns of predicted external correlates, and (d) convergence between self and observer ratings.
- Perceived Stress Scale (PSS): This 10-item scale is the most widely used psychological instrument for measuring the perception of stress. It is a measure of the degree to which situations in one’s life are appraised as stressful. Items were designed to assess how unpredictable, uncontrollable, and overloaded participants find their lives.
- Comorbidity Questionnaire for MS: This measure asks the participants to self-report the presence or absence of 36 comorbidities that are frequent in the MS or general population.
- Patient Determined Disability Steps (PDDS): This 1-item scale is a simple and reproducible measure to evaluate disease progression in MS. Participants select one of eight responses that best matches their current level of disability. Higher scores indicate higher levels of disability.
- Pet Ownership Questionnaire: This measure asks participants to self-report the presence or absence of pets, length of pet ownership, and the type of pets owned.
- Stop-Bang Questionnaire: This 8-item screening instrument assesses characteristics known to confer risk for Obstructive Sleep Apnea. Scores ≥3 indicate elevated risk for sleep apnea. Reliability and validity data support clinical utility of this measure.
- painDETECT Questionnaire: This measure assesses the presence and nature of neuropathic pain symptoms. Questions address the quality of neuropathic pain symptoms, pain course pattern, and radiating pain.
- Nociplastic pain – American College of Rheumatology 2016 Fibromyalgia Survey Criteria: This measure assesses the number of painful body regions using the Michigan Body Map (0-19) and includes questions about related symptoms such as problems with thinking, fatigue, and sleep (0-12). A resulting continuously scaled metric (possible range 0-31) can be used as a self-reported proxy index for central sensitization. This measure has been previously used to quantify centralized pain in other clinical populations and relates strongly to functional neuroimaging findings in nociplastic pain and is a robust predictor of both pain and disability.
- Compensatory Cognitive Strategies Scale: This 24-item measure assesses cognitive strategies, including of memory functioning and executive functioning, used in daily life. Each cognitive strategy is rated on a 5-point frequency scale, with a total score ranging from 0 to 96. Higher scores indicate more frequent use of the cognitive strategies.
- 1-Month Recall Falls/ Near Falls: This 2-item measure assesses if and how many falls and/or near falls occurred in the past month.
- Falls Efficacy Scale (FES-I): This 16-item scale assesses concerns about falling while doing a range of basic to demanding activities, both physical and social. Total score can range from 16 to 64. Higher scores indicating greater concern about falling.
- Fear of Falling Avoidance Behavior Questionnaire (FFABQ): This is a 14-item measure assesses avoidance of specific activities due to fear of falling. Higher scores indicate greater level of activity limitations and restrictions due to fear of falling.
- Concern and Fear of Falling Evaluation (CAFFE): This 27-item measure assesses concern about falling and fear of falling.
