# Supplementary material for: Optimizing Detection and Prediction of Cognitive Function in Multiple Sclerosis With Ambulatory Cognitive Tests: Protocol for the Longitudinal Observational CogDetect-MS Study
Source: JMIR Res Protoc. 2024 Sep 26;13:e59876. doi: 10.2196/59876 (PMC11467611; doi:10.2196/59876)
Supplement: Multimedia Appendix 2 [file resprot_v13i1e59876_app2.docx]

**Motor Function**:

- The NIHTB 9-Hole Pegboard Dexterity Test: This test assesses fine motor dexterity, specifically the ability to coordinate the fingers and manipulate objects in a timely manner. Participants place 9 pegs into a pegboard with 9 holes, then remove them as quickly as possible. One timed trial is done with each hand.
- The NIHTB Grip Strength Test: This test assesses upper extremity strength. Participants squeeze a hand dynamometer as hard as they can with each hand.
- The 4-Meter Backward Walk Test (gait speed): this test assesses balance and fall risk. Participants walk backward a short distance at a normal speed on a flat surface. Scores are recorded as time required to walk 4 meters on each of two trials, with the better trial used for scoring.
- The NIHTB 4-Meter Walk Gait Speed Test: This test assesses locomotion. Participants walk a short distance at their usual pace on a flat surface. Scores are recorded as time required to walk 4 meters on each of two trials, with the better trial used for scoring.
- The NIHTB 2-Minute Walk Gait Endurance Test: This test assesses the ability to sustain effort that requires conjoint work capacities from cardiopulmonary, biomechanical, and neuromuscular function. Participants walk as quickly as possible for 2 minutes on a flat surface. The score is recorded as the total distance walked during a 2-minute period.
- The NIHTB Standing Balance Test: This test assesses the ability to orient the body in space and maintain an upright posture without falling. Participants complete a series of poses on the ground and on a foam pad, with eyes open and eyes closed.

**Cognitive Function**:

- The NIHTB Picture Vocabulary Test: This test assesses receptive vocabulary and is administered in a computer-adaptive test format. Participants determine which of four pictures best matches the meaning of a word presented auditorily.
- The NIHTB Flanker Inhibitory Control and Attention Test: This test uses algorithms of accuracy and reaction time to assess executive functioning, inhibitory control, and attention. Participants focus on a given visual stimulus while inhibiting attention to the stimuli flanking it.
- The NIHTB List Sorting Working Memory Test: This test assesses the ability to store information until the amount of information to be stored exceeds one’s capacity to hold that information. Participants recall and sequence various stimuli that are presented both visually and auditorily.
- The NIHTB Dimensional Change Card Sort (DCCS) Test: This test assesses cognitive flexibility and attention. Participants match a target picture to a series of picture pairs according to shape or color.
- The NIHTB Pattern Comparison Processing Speed Test: This test assesses the amount of information that can be processed within a certain unit of time. Participants are asked to quickly identify whether two visual patterns are the same or not the same.
- The NIHTB Picture Sequence Memory Test: This test assesses episodic memory and the cognitive processes involved in the acquisition, storage, and retrieval of new information. A sequence of pictures describing an activity is visually and auditorily presented, and participants are asked to remember and place the pictures in the same order initially presented.
- The NIHTB Oral Reading Recognition Test: This test assesses reading decoding skills and crystallized abilities. Participants are asked to read and pronounce letters and words as accurately as possible.
- The NIHTB Oral Symbol Digit Test: This test assesses processing speed. Participants match numbers with symbols according to a key at the top of the page. The key has a series of nine symbols, each of which is paired with a single number, labelled 1-9. Participants verbally state the digit associated with each of these symbols as quickly as possible. The score is recorded as the number of correct items completed in 120 seconds, with a maximum of 144 correct items.
- The Rey Auditory Verbal Learning Test (RAVLT): This test assesses verbal learning and delayed recall. Participants are read a list (List A) of 15 common, but unrelated, words and asked to verbally recall as many of the words as possible. This process is repeated five times. Following the fifth trial, a second list of 15 words (List B) is read and participants are asked to verbally recall as many of the words as possible. Following this interference trial, participants, without repetition of List A, are again asked to verbally recall the words from List A. After a delay of approximately 30-minutes, participants are asked to freely recall as many of the 15 words from List A as they can. Finally, participants are read a longer list of 52 words and asked to verbally identify the words from List A. The scores collected include the number of words recalled on the delay trial, as well as the number of true and false positives on the recognition portion of the test.
- Paced Auditory Serial Addition Test - 3 second (PASAT): This test assesses sustained attention, speed of information processing, and working memory. An audio recording presents a single digit number every three seconds, and participants are asked to add each consecutive digit to the one immediately preceding it and say each sum aloud. A practice series of 11 digits (10 answers) precedes the test. There are a total of sixty-one digits presented with a maximum of 60 correct answers.
- The Symbol Digit Modality Test (SDMT): This test assesses attention and processing speed. The oral version of this measure is used, given the high rate of motor interference in people living with MS. The test form presents a series of nine symbols, each of which is paired with a single digit, labelled 1-9, in a key at the top of the form. The remainder of the form has a pseudorandomized sequence of the symbols, and participants verbally state the digit associated with each of these symbols as quickly as possible. The score is the number of correct answers in 90 seconds.
- ReacStick Test: This test assesses cognitive deficits—such as deficits in attention, processing speed, and inhibitory control—using a motor test called the ReacStick. The ReacStick is an instrument that looks like a yard stick with a small box at the end that records time. Participants are asked to use the hand that they use the most; for most participants this is their dominant hand. From a seated position, participants rest their forearm on a table with their hand extended beyond the table edge. Participants are instructed to position their hand around the box of the ReacStick (held by the test examiner) without grasping it. Then the examiner drops the ReacStick. The ReacStick test assesses both simple reaction time and complex (recognition) reaction time. Simple reaction time is the measure of the time (in milliseconds, m/s) it takes for the participant to grasp (or tap, if they are unable to grasp) the ReacStick after it is dropped. Each simple reaction time session consists of 4 practice trials and 10 data collection trials. The mean score in m/s is used. During complex reaction time sessions, light-emitting diodes on the box illuminate randomly during 50% of the trials at the instant the device is dropped. Participants are instructed to catch the falling ReacStick only during the trials in which the light-emitting diodes illuminate, and to resist catching it when the diodes do not illuminate, consistent with a standard go/no-go testing paradigm. During complex reaction time sessions, verbal instructions emphasize response accuracy, not speed. Participants complete 6 complex reaction time practice trials and 20 data collection trials. Reaction time and accuracy are recorded. Reaction time accuracy is recorded as the percentage of correctly performed trials/ total number of trials.
