# Supplementary material for: Optimizing Detection and Prediction of Cognitive Function in Multiple Sclerosis With Ambulatory Cognitive Tests: Protocol for the Longitudinal Observational CogDetect-MS Study
Source: JMIR Res Protoc. 2024 Sep 26;13:e59876. doi: 10.2196/59876 (PMC11467611; doi:10.2196/59876)
Supplement: Multimedia Appendix 3 [file resprot_v13i1e59876_app3.docx]

- Perceived cognitive function is assessed with three items (slow thinking, foggy thinking, and ability to remember) rated on a 0-100 numerical rating scale (NRS). Scores on the three items are averaged for an overall perceived cognitive functioning score.
- Pain intensity is assessed with the item: “How much pain do you have right now?” on a 0–100 NRS.
- Fatigue intensity is assessed with two questions: “How much PHYSICAL fatigue do you have right now?” and “How much MENTAL fatigue do you have right now?” on a 0-100 NRS.
- Perceived stress is assessed with the item: “How would you rate your current amount of stress?” on 0-100 NRS.
- Depressed mood is assessed with three items (sad, hopeless, discouraged) rated on a scale from 0 (not at all) to 4 (extremely).
- Sleep quality is assessed once each morning with two questions: “How refreshed did you feel when you woke up this morning?” and “How would you rate the quality of your sleep?” each on a 0-100 NRS. Participants also enter their sleep and wake times.
- Social participation is assessed once per day at the last assessment of the day using items from the NeuroQoL Ability to Participate in Social Roles and Activities item bank, adapted for daily use by adding an item stem, “Today,” and changing item word tense.
- Physical function is assessed once per day at the last assessment of the day with items that assess global lower and upper extremity functioning: “Today” … “How much difficulty do you have with your mobility, that is your ability to get around?” and “How much difficulty do you have with using your hands to complete tasks?” rated on a 0-100 NRS.
- Falls are assessed at the first and last assessments of the day, using a previously developed falls diary method in MS. Falls are defined as “a slip or trip where you lost your balance and landed on the floor or ground or lower level.” At the first assessment of the day, we ask: “Did you fall overnight?” Response options are “Yes/No” with branching logic, such that if the participant selects “Yes”, they are prompted to enter “How many falls?” and subsequently “What time?” with a dropdown list and “Were you distracted or doing two things at once when you fell?” for each fall indicated. At the final assessment of the day, we ask: “Did you have any falls today?” with the same branching logic as the morning assessment.
- Environmental distractions are assessed with the questions: “During the brain games, were you distracted by anything in your environment?” Response options are “Yes/No”, with branching logic, such that if the participant selects “Yes”, they are offered the following checklist with instructions to “please check all applicable distractions” 1) distracting sounds; 2) distracting lights; 3) distracting temperature; 4) social distractions; 5) physical distractions (pain, hunger, etc.); 6) other distraction.
- Substance use is assessed with the question: “Since your last assessment, did you consume (check all that apply): 1) alcohol; 2) caffeine; 3) nicotine; 4) cannabis; 5) narcotic/opioid drug; 6) other prescription drug; 7) other over the counter drug; 8) other recreational drug.
- Location during tests is assessed with the question: “Where are you?” 1) inside my home; 2) inside at work; 3) outdoors; 4) other.
- Activity pacing is assessed at each time point, except the morning assessment, with three items: “Since you last provided ratings, did you: Take rest breaks during activities?; Go slower and spread out activity over time?; Break up activity into manageable pieces?” Each item is rated on a scale from 0 (Never) to 4 (Always).
